# Monitoring Crimean-Congo haemorrhagic fever virus RNA shedding in body secretions and serological status in hospitalised patients, Turkey, 2015

**DOI:** 10.2807/1560-7917.ES.2020.25.10.1900284

**Published:** 2020-03-12

**Authors:** Dilek Yagci-Caglayik, Bircan Kayaaslan, Derya Yapar, Aysel Kocagul-Celikbas, Aslinur Ozkaya-Parlakay, Mestan Emek, Nurcan Baykam, Hasan Tezer, Gulay Korukluoglu, Aykut Ozkul

**Affiliations:** 1 Marmara University Pendik Training and Research Hospital, Department of Infectious Diseases and Clinical Microbiology, Istanbul, Turkey; 2 Public Health General Directorate of Turkey, Virology Laboratory, Ankara, Turkey; 3 Ankara University Faculty of Veterinary Medicine, Department of Virology, Ankara, Turkey; 4 Yıldırım Beyazıt University Faculty of Medicine, Department of Infectious Diseases and Clinical Microbiology, Ankara, Turkey; 5 Hitit University Faculty of Medicine, Department of Infectious Diseases and Clinical Microbiology, Corum, Turkey; 6 Health Sciences University, Ankara Children's Hematology Oncology Training and Research Hospital, Department of Pediatric Infectious Diseases, Ankara, Turkey; 7 Akdeniz University Faculty of Medicine, Department of Public Health, Antalya, Turkey; 8 Gazi University Faculty of Medicine, Department of Pediatric Infectious Diseases, Ankara, Turkey; 9 Ankara University, Biotechnology Institute, Ankara, Turkey

**Keywords:** Crimean–Congo haemorrhagic fever virus, CCHF, viral shedding, antibody response, urine, faeces, genital swab, serum, RT-PCR

## Abstract

**Introduction:**

Crimean-Congo haemorrhagic fever (CCHF) is a tick-borne disease in Africa, Asia, the Balkan peninsula, the south-east of Europe and the Middle East, with mortality rates of 3–30%. Transmission can also occur through contact with infected animals or humans.

**Aim:**

This observational, prospective case series aimed to investigate detectable viral genomic RNA in whole-body fluids and antibody dynamics in consecutive daily samples of patients diagnosed with CCHF until discharge from hospital.

**Methods:**

We tested 18 patients and 824 swabs and sera with RT-PCR and 125 serum samples serologically.

**Results:**

The longest duration until clearance of viral RNA was 18 days from serum collection and 18, 15, 13, 19 and 17 days, respectively, from nasal, oral, genital (urethral or vaginal) and faecal swab, and urine. In seven patients, viral load decreased in serum at the same time as it increased in urine or persisted at the same logarithmic values. Despite clearance in serum, viral RNA was detected in faeces and genital swabs in two and three patients, respectively. Viral clearance from body fluids occurred earlier than from serum in eight patients on ribavirin treatment. The shortest seroconversion time was 3 days after symptom onset for IgM and IgG. Seroconversion of IgG occurred until Day 14 of symptoms.

**Conclusion:**

We report persistence of viral RNA in urine, faeces and genital swabs despite serum clearance. This may indicate a need for extending isolation precautions, re-evaluating discharge criteria and transmission risk after discharge, and considering oral swabs as a less invasive diagnostic alternative.

## Introduction

Crimean–Congo haemorrhagic fever (CCHF) is a vector-borne disease seen in Africa, Asia, the Balkan peninsula, south-eastern Europe and the Middle East and causing outbreaks with fatality rates up to 83% [1]. The case fatality rates reported in outbreaks of the Europe clades of the virus were 3–100%, 15–83% for the Asia clades and 0–75% for the Africa clades [1]. Infection in humans occurs by tick bite or contact with the blood of infected animals or CCHF patients. The main tick vector for CCHFV is the *Hyalomma* genus, but *Rhipicephalus* and *Dermacentor* species are also reported as vectors [1,2]. The CCHF virus (CCHFV) is a negative-sense RNA virus in the *Nairoviridae* family within the order *Bunyavirales*. CCHFV has three genomic segments, small, medium and large, that encode the nucleoprotein, glycoproteins and RNA-dependent RNA-polymerase, respectively. 

In the World Health Organization (WHO) European Region, Albania, Armenia, Bulgaria, Georgia, Greece, Kosovo*, Russia, Serbia, Turkey and Ukraine report outbreaks caused by the Europe clades [3,4]. Two cases were reported from Spain in 2016, one fatal and the other nosocomial, both were caused by the Africa clade. In 2018, another fatal case was reported in Spain close to the border of Portugal [5,6]. In Turkey, 874 cases per year (range: 343–1,318) were reported in the last decade [7]. More than 10,000 confirmed cases have been reported in Turkey since 2002, with a fatality rate of 4.6% [8].

Besides known transmission routes of CCHFV, there are molecularly undocumented case reports indicating that transmission may occur via sexual contact or from using the same toilet or room [9-11]. The earliest antibody response to CCHFV has been reported on Day 4 and 5 after onset of the symptoms or Day 2 to 6 after hospitalisation [12-14].

Standard precautions are recommended in the follow-up of non-haemorrhagic CCHF patients but if haemorrhage exists, contact and droplet precautions are recommended [15]. Because high-level infectious disease units are lacking in endemic countries, cohorting confirmed cases is the usual method during follow-up [16,17]. There are no recommendations for CCHF patients after discharge from hospital or in home care settings [18].

In this study, in order to contribute to control and prevention strategies, we aimed to monitor viral RNA shedding as an indicator of the infectiousness of various body fluids and secretions as well as the antibody dynamics by daily sampling of CCHF patients during their hospitalisation.

## Methods

This study was an observational, prospective case series conducted only from 15 June to 15 July 2015 because of the limited budget of the project and the schedule of the virology laboratory of the Public Health Directorate in Ankara (formerly Refik Saydam National Public Health Agency).

In Turkey, CCHF patients are followed in the hospitals to which they are admitted. However, if there is a need for blood components, patients are sent to the nearest reference tertiary hospitals in one of the 18 provinces (one of which is Ankara) as decided by the Turkish Ministry of Health in 2011. Five tertiary reference hospitals in Ankara were accepting CCHF patients from neighbouring provinces in the year the study was done. Çorum had been a province sending complicated CCHF patients to the reference hospitals in Ankara but a university hospital was established in Çorum in 2014 after which patients were followed there. Considering the easy and rapid transfer of samples across the short distance between the hospitals and the virology laboratory, tertiary reference hospitals in Ankara and Çorum University Hospital were asked to participate in the study. Voluntary infectious disease and clinical microbiology specialists from three tertiary reference hospitals in Ankara and one in Çorum sent consecutive samples from CCHF patients to the virology laboratory at the Public Health Directorate.

### Patients and samples

We included in the study 19 patients who tested positive by the virology laboratory of the Public Health General Directorate with the RealStar CCHFV RT-PCR Kit 1.0 (Hamburg, Germany). There were no exclusion criteria. However, one patient was excluded because of inappropriate sampling. Starting from the day CCHF was diagnosed, seven types of body fluids and secretions including serum, nasal swabs (from two nostrils), oral swabs (buccal mucosa and sublingual), axillary swabs (from the skin of two axillary fossae in order to obtain sweat gland secretion), urine, faecal swabs and urethral or vaginal swabs were collected from the 18 hospitalised individuals daily until discharge to measure the duration of viral RNA shedding and provide data on the antibody response dynamics.

All samples were transported daily to the virology laboratory in Copan Transystem virus transport medium (Brescia, Italy) in a cold chain. No vaginal or urethral swabs were taken from patients younger than 18 years.

### Molecular and serological tests

Viral RNA was extracted (EZ1 Advanced XL-QIAGEN; Hilden, Germany) and analysed using a quantitative one-step real-time RT-PCR with primers and probes targeting the S segment. The Rotor-Gene Probe RT-PCR kit (QIAGEN, Hilden, Germany) and Rotor-Gene Q platform were used. Viral load was measured with the intra-assay standards as described before [19].

The presence of IgG and IgM antibodies against CCHFV in serum was detected using an indirect enzyme-linked immunosorbent assay (ELISA) (Vector-Best, Novosibirsk, Russia). ELISA tests were completed in two sessions. According to the manufacturer’s instructions, optical densities (OD) > 0.214 and > 0.250 for IgM and IgG, respectively, were considered as positive results in the first session and > 0,214 and > 0,240 for the second. Higher OD values that could not be read by the ELISA reader were taken as 3.0. All tests were performed at the virology laboratory of the Public Health Institute of Turkey.

### Statistical analysis

Statistical analysis was performed using SPSS v.18.0. Descriptive statistics were considered as mean, standard deviation, median, minimum and maximum for quantitative variables, and as number and percentage of categorical variables. In statistical comparisons, the Spearman Rank correlation (viral load, viral RNA clearance and severity scoring index) was used for comparison of viral load at the beginning and viral RNA clearance in serum. A Mann–Whitney U test (haemorrhage, viral load at the beginning) was used when evaluating the association between haemorrhage and viral load. A scatter graph was constructed to show the antibody response on follow-up.

In case management of CCHF, a clinical severity scoring index is defined in adult patients for prognosis and the prediction of death. According to platelet count, prothrombin time, fibrinogen level, the existence of bleeding and somnolence symptoms, patients are categorised as mild, moderate or severe [20].

If a measurement was missed, we used instead the value of the measurement recorded on the following day. If the missing values were on the last day of the follow-up, the values recorded on the day before were taken as the last values.

### Ethical statement

Ethical Approval was obtained for CCHF reference hospitals in Ankara and Çorum. This study was approved by the Clinical Trials Ethics Committee, Faculty of Medicine, Ankara University under approval number 11–455–15. Informed consent was obtained from all participants or from the parents of those younger than 18 years.

## Results

### Clinical characteristics of the patients

There were 12 male and six female patients with a mean age of 45 (standard deviation (SD): 20.6 years; range: 7–73 years). Of 18 patients followed up, 15 were adults and three were children. Paediatric patients were 7-, 14- and 15-years-old. Haemorrhage was seen only in five of 18 patients at any time during the follow-up. According to the clinical severity scoring index for the prognosis and therapy of CCHF, only one patient had severe disease, 11 patients had moderate and three had mild disease. The number of patients treated with ribavirin was 11, three children and eight adults. The mean starting day of monitoring after the onset of symptoms was 5.06 (SD: 2.24 days; range: 1–10 days). The mean duration of hospitalisation was 5.83 (SD: 2.64 days; range: 2–10 days). All patients were discharged from the hospital.

Of the 18 patients, 14 had a history of tick bites. Among those 14, the incubation period from the bite to the onset of symptoms varied from null to 26 days. The mean incubation time was 4.86 days; the median was 4 days.

### Viral load monitoring in body fluids and secretions

We collected 824 samples of eight different sample types from 18 patients. Seventy-six samples could not be properly collected on the expected day and were discarded (Table 1).

**Table 1:** Crimean-Congo haemorrhagic fever patients (n = 18) and sample types (n = 130), Turkey, 2015

Sample type	Number of patients	Total follow-up patient-days	Number of collected samples	Number of days without sample collection
Serum for molecular analysis	18	130	120	10
Nasal swab	18	130	117	13
Oral swab	18	130	120	10
Axillary swab	1	10	10	0
Urine	18	130	118	12
Vaginal swab	6	39	37	2
Urethral swab	9	71	58	13
Faecal swab	18	130	118	12
Serum for serology	18	130	126	4

Serum viral loads ranged from 0 to 1.59×10^8^ copies/mL. During the follow-up period, the viral loads of the patients declined. The longest duration of viraemia was 17 days and viral RNA clearance, or undetectable CCHFV RNA, was recorded on Day 18 in this patient (Supplementary Table S15). The longest viral RNA clearance periods from nasal swab, oral swab, urine, genital (urethral or vaginal) swab and faecal swab were, respectively, 18, 15, 17, 13 and 19 days, in different patients (Supplementary Tables S15, S17, S18). One sweat sample was taken and was positive in RT-PCR only on Day 15 and Day 19 during follow-up of one patient (Supplementary Table S15). In seven patients, we observed the viral load in serum samples decrease, while at the same time, the load in urine samples increased or persisted at the same logarithmic values (Supplementary Table S1, S3, S5, S13, S16-18).

Ribavirin treatment was started in three paediatric patients before the first day of hospitalisation (Day 3 and Day 4 after symptom onset) and viral RNA clearance in the serum was obtained on Day 6 after symptom onset in the child who took the longest (Supplementary Tables S9-11). For eight adult patients, ribavirin treatment was started between Day 1 and Day 10 after symptom onset. Viral RNA clearance in serum was reached earliest on Day 3 and latest on Day 18 after symptom onset (Supplementary Tables S9, S15) (Table 2). In these eight patients, clearance of viral RNA from body fluids occurred earlier than clearance from serum samples (Supplementary Tables S6, S8-12, S14, S16) (Table 2).

**Table 2:** Viral RNA clearance days in ribavirin-treated patients with Crimean-Congo haemorrhagic fever, Turkey, 2015 (n =11)

Patient code	Ribavirin start (day after symptom onset)	Viral RNA clearance (day after symptom onset)						
		Serum	Nasal	Oral	Urine	Vaginal	Urethral	Faecal
C1	1	< 3	< 3	< 3	< 3	NA ^a^	Not tested	< 3
C3	1	< 4	< 4	< 4	< 4	NA ^a^	Not tested	5
C2	1	6	5	4	< 3	NA ^a^	Not tested	< 3
H3	2	5	5	5	< 3	NA ^b^	< 4	< 4
Y6	3	< 6	< 5	< 5	< 6	NA ^b^	< 7	< 5
H1	4	8	< 5	6	< 5	NA ^b^	< 5	< 5
H2	4	> 14	11	10	> 14	14	NA ^c^	14
H5	5	13	12	8	> 13	13	NA ^c^	12
N1	6	> 9	9	8	> 9	NA ^b^	> 9	> 9
N2	6	> 9	> 9	8	7	7	NA ^c^	< 6
H4	10	18	18	15	12	NA ^b^	11	19

NA: not applicable.

^a^ Not applicable because they were children.

^b^ Not applicable because they were male.

^c^ Not applicable because they were female.

In the patients who started ribavirin treatment on Day 1 or 2 after symptom onset (Supplementary Tables S6, S9, S10, S11, S14), viral RNA shedding from body fluids and sera was significantly shorter than in those started on Day 4, 5 and 6 (Supplementary Tables S8, S12, S13, S16) (Table 2). 

No correlation was detected between viral load and the severity score index of the patients with the Spearman Rank correlation. However, there was a significant association (r = 0.71 and p < 0.05) between initial serum viral load and duration viral RNA shedding when analysed with the Spearman Rank correlation (Supplementary Table S19).

Serum viral loads of the haemorrhagic patients were significantly higher (p < 0.05; median: 2.5×10^7^ copies/mL; mean: 5.3×10^6^ copies/mL; range: 1.2×10^5^–1.6×10^8^ copies/mL) than non-haemorrhagic patients (median: 1.9×10^3^ copies/mL; mean: 2.9×10^5^ copies/mL; range: 0–3.5×10^6^ copies/mL) in the Mann–Whitney U test (Supplementary Table S20).

### Serological monitoring

**Figure 1:**
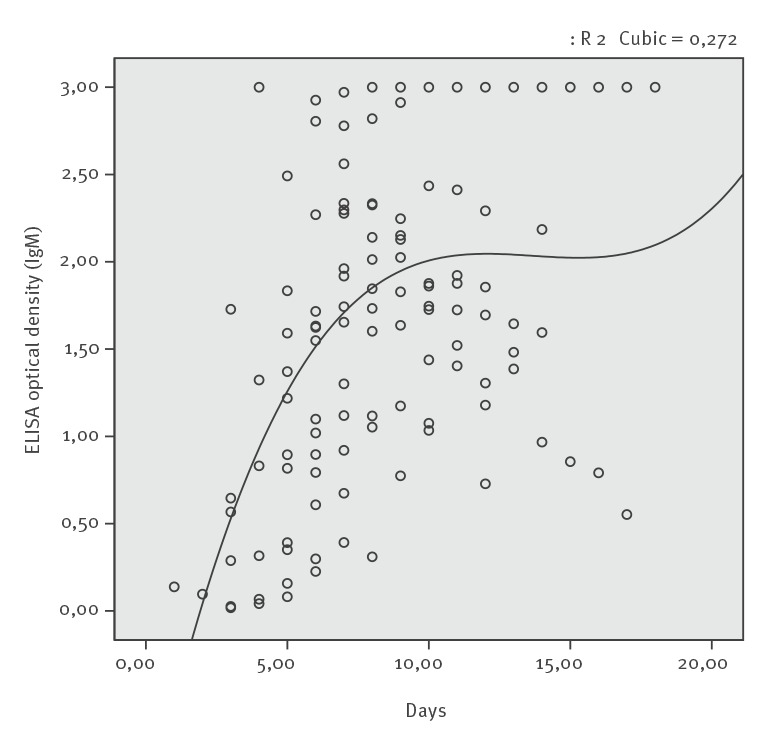
IgM by days after symptom onset, Crimean-Congo haemorrhagic fever, Turkey, 2015 (n = 18)

**Figure 2:**
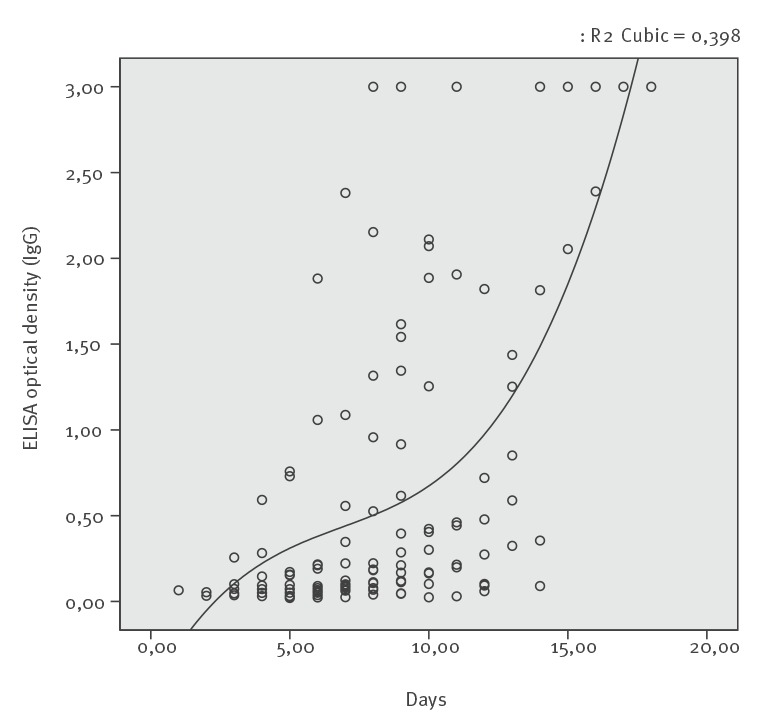
IgG by days after symptom onset, Crimean-Congo haemorrhagic fever, Turkey, 2015 (n = 18)

The shortest seroconversion times were 3 and 4 days after the onset of symptoms for anti-CCHFV IgM and IgG, respectively (Supplementary Tables S2, S10). Seroconversion of anti-CCHFV IgM was detected in all patients within 10 days. In the patients who seroconverted, anti-CCHFV IgG positivity occurred until Day 14 after symptom onset, while in five patients, IgG seroconversion was not detected (Figure 1, Figure 2), (Supplementary Tables S4, S6, S8, S9, S12).

 Anti-CCHFV IgM and anti-CCHFV IgG responses were detected at the earliest on Days 3 and 4 and at the latest on Days 6 and 14 of the illness, respectively.

With daily measurement, the decline of serum CCHFV RNA concentrations and increase in anti-CCHV IgG optical density values happened concomitantly in 12 patients. (Supplementary Tables S2, S3, S5, S7, S8, S10, S12-16, S18).

## Discussion

CCHF is an important public health problem with high mortality rate and secondary attack rates. Known transmission routes are tick bites and contact with the blood and body secretions of an infected person. Consequently, detection of body secretions with high risk is going to be important in the prevention of transmission. Detecting viral RNA shedding is an objective method that can be used to explore the transmission of viruses. Understanding transmission ways enables individuals to be protected from the disease. Training the public in prevention can help prevent this viral disease.

To our knowledge, this is the first study monitoring CCHF viral loads in various body fluids and secretions collected on consecutive days during the acute phase of infection. Previous studies have been cross-sectional and report CCHFV RNA positivity in urine on Day 7, 19 and 25 after symptom onset [21,22]. In our study, initial levels of viral load and days until clearance of viral RNA were associated. We observed that patients with high viral loads shed viral RNA for a longer period, therefore isolation precautions may need to be extended.

Using molecular methods, we found that viral RNA shedding from body fluids other than serum continued although serum samples of the patients were PCR-negative. However, in Turkey, the decision to discharge CCHF patients from hospital is based on clinical improvement or negative CCHFV PCR results in serum [16,17]. Discharge criteria may need to be examined based on the data provided in this study.

Anti-CCHFV IgM was detected in the first and anti-CCHFV IgG in the second week after symptom onset. These findings are in line with an 11-year follow-up study in South Africa that included 101 patients, but did not consecutively investigate them [14]. Anti-CCHFV IgM antibodies appeared in parallel with the decline in viral load in 12 patients. It has been noted that anti-CCHFV IgM may not be detected in severe and fatal cases but in our study, all patients had an IgM response [14].

None of our patients had a viral load reaching the threshold value associated with fatality, which is reported to be ca 1×10^9^ copies/mL, and none of our patients died [13,23]. The viral loads in the genital and faecal swabs of seven patients were higher than in serum samples taken simultaneously, and viral RNA was detectable until Day 13 after symptom onset. There are reports suspected sexual transmission 2 days before to 12 days after the onset of the symptoms. One of these cases was discharged from the hospital on Day 8 after symptom onset and 3 or 4 days later (12 days after the onset of symptoms) had sexual contact with his partner, who was diagnosed with CCHF 7 days later [10]. The other example of sexual transmission occurred on Day 10 after disease onset in a case without haemorrhagic symptoms [9]. Our study suggests that sexual contact can be a transmission route for CCHFV because of the presence of viral RNA in genital samples as late as 13 days after symptom onset. In our opinion, it is advisable to inform patients about the ongoing risk of transmission after discharge from hospital.

Detection of viral RNA in oral swab, nasal swab, urine, faecal swab and sweat swab samples until, respectively, Day 14, 17, 16, 18 and 19 after onset of symptoms could indicate the need for droplet and contact precautions around CCHF patients. Droplets may have been the route of transmission in a suspected nosocomial transmission in Russia to a healthcare worker who had changed the bedsheets and cleaned the patient’s room [24].

During the initial days of follow-up, serum samples and oral swabs were PCR-positive on the same days. Obtaining oral swabs may therefore be considered as a less invasive alternative for molecular diagnosis than taking blood samples.

At the time of viral RNA clearance from the serum samples of four patients, viral RNA shedding in urine was continuing. In addition, while the viral load in serum samples was decreasing in 10 patients, the viral load was increasing or remaining at the same log levels in the urine samples. This situation can be considered as the urinary phase of CCHF, which is also seen in other viral haemorrhagic diseases. There are case series for yellow fever, dengue, Zika and West Nile virus, all of which are in the *Flavivirus* family, that report serum samples as negative, but urine positive for the viruses at the same time [25-28]. The kidney has been found to be one of the organs with high viral loads of CCHFV [29,30].

The presence of CCHFV RNA in urine and faeces is an important observation for viral pathogenesis and transmission. There was a case in 2006 where the only suspected risk factor was sharing the same toilet and room for 5 days with a patient diagnosed with CCHF urinary infection [11]. Data from our study and future investigations into viral RNA shedding may provide evidence for re-examining isolation precautions.

That starting ribavirin treatment within 3 days after onset of symptoms is effective and prevents transmission by shortening the period of viral RNA shedding is a hypothesis that we considered to have with the molecular method in this study [20].

On the days when serum samples were PCR-negative, viral RNA shedding could nevertheless be detected in the nasal swabs of two patients, the genital swabs of three patients, the faecal swabs of two patients and the urine samples of four patients. Unidentified exposure, which excludes needlestick injury and contact with body fluids, occurred in 13.7% of nosocomial CCHFV infections in Turkey [31]. Contact with contaminated clothes and sheets or handling patients or body fluids caused nosocomial CCHF in İran [32]. In addition to standard precautions, additional contact and droplet precautions may be considered in the follow-up of CCHF inpatients, even when haemorrhage is not present. We could not find any recommendations for CCHF in home care settings but based on our data, it may be appropriate to continue standard, contact and droplet precautions [18]. After discharge, it may be advisable that the patients do not have sexual contact to prevent transmission until Day18 after the onset of symptoms.

In this study, we investigated viral RNA in the samples rather than whole virus. This does not equal infectiousness, and constitutes a major limitation. There is no study on CCHFV examining and comparing the real infectiousness with molecular methods and cell culture, but in a case report for Ebola virus, cell culture negativity, which means non-infectivity, started 3–5 days before RNA clearance from the samples [33]. It is not clear that the RNA positivity means infectivity and causes transmission but it may indicate the presence of the competent virus. 

We did not investigate correlations between viral loads in body fluids and age, sex and underlying conditions because there was no homogeneity in the patients’ starting day of treatment, starting day of follow-up and duration of follow-up. In addition, the number of patients was limited.

The limitations of this study are as follows: (i) patient follow-up periods were not the same because they were hospitalised on different days of symptoms; (ii) a standard follow-up time period could not be set and patients could not be followed up after serum viral RNA clearance because of the discharge procedures; (iii) actual viral shedding can only be detected with the isolation of the virus in cell culture but because high containment laboratory conditions (BSL-4) are required for CCHFV cultural isolation, viral shedding was detected only with a molecular method; (iv) the number of patients included was limited.

The strength of this follow-up study was the molecular investigation of CCHFV in different body fluids and secretions and sera at the same time. To our knowledge, this is the first study analysing the risk and duration of CCHFV RNA and the data provide insight into the pathogenesis of CCHFV, showing virus shedding based on molecular laboratory evidence. We believe that a revision of isolation precautions in the guidelines would be beneficial.

## *Note

*This designation is without prejudice to positions on status, and is in line with United Nations Security Council Resolution 1244/99 and the International Court of Justice Opinion on the Kosovo declaration of independence.

## Supplementary Data

192000 Supplement
