# Supplementary material for: Monitoring Crimean-Congo haemorrhagic fever virus RNA shedding in body secretions and serological status in hospitalised patients, Turkey, 2015
Source: Euro Surveill. 2020 Mar 12;25(10):1900284. doi: 10.2807/1560-7917.ES.2020.25.10.1900284 (PMC7078823; doi:10.2807/1560-7917.ES.2020.25.10.1900284)
Supplement: Supplement [file 19-00284_YAGCI-CAGLAYIK_Supplement.pdf]

PATIENTS' VIRAL LOADS AND ANTIBODY RESPONSES

This supplementary material is hosted by *Eurosurveillance* as supporting information alongside the article "Monitoring Crimean-Congo Haemorrhagic Fever Virus RNA Shedding in Body Secretions and Serological Status in Hospitalised Patients, Turkey, 2015" on behalf of the authors who remain responsible for the accuracy and appropriateness of the content. The same standards for ethics, copyright, attributions and permissions as for the article apply. Supplements are not edited by *Eurosurveillance* and the journal is not responsible for the maintenance of any links or email adresses provided therein.

	7	8	9	19
Serum	9,92E+01	7,82E+01	2,86E+01	Negative
Nasal	1,79E+02	4,89E+03	1,52E+01	Negative
Oral	9,85E+02	Negative	Negative	Negative
Urine	1,25E+02	1,48E+02	1,25E+02	Negative
Vaginal*	3,45E+04	2,74E+04	2,14E+03	Negative
Fecal	3,17E+02	1,42E+03	2,93E+03	Negative
IGM	2,97	3,00	3,00	3
IGG	0,56	3,00	0,92	2,11

Table-S1: Y1 coded patient (viral loads in copies/mL and OD values on monitored days) *Vaginal viral load is higher than urine and serum. Viral load of urine is higher than serum on follow-up days.

	1	2	3	4	5	6	7	8	9	10	11
Serum*	2,58E+05	1,39E+05	7,47E+04	4,93E+03	1,07E+03	5,46E+01	3,08E+00	Negative	Negative	Negative	Negative
Nasal	9,59E+02	5,12E+02	N/A	5,36E+01	2,41E+03	7,21E+02	6,08E+01	8,04E+00	Negative	Negative	Negative
Oral	2,24E+02	2,46E+02	N/A	6,08E+02	1,08E+01	1,00E+00	Negative	Negative	Negative	Negative	Negative
Urine	Negative	Negative	N/A	Negative	2,73E+01	1,99E+01	Negative	1,27E+01	Negative	Negative	Negative
Urethral	Negative	Negative	N/A	4,65E+01	1,43E+01	Negative	Negative	Negative	1,96E+00	Negative	Negative
Fecal	Negative	Negative	N/A	Negative	2,67E+01	Negative	Negative	Negative	Negative	Negative	Negative
IGM	0,138	0,096	0,288	0,567	1,371	2,805	2,779	2,333	2,247	1,861	1,876
IGG	0,065	0,033	0,036	0,072	0,172	0,211	0,347	0,957	1,542	1,886	3

Table-S2: Y2 coded patient (viral loads in copies/mL and OD values on monitored days) (N/A: not sent to the laboratory) *There is viral clearance in serum on 8th day, but not in urine and not urethral swab on the 9th day. IgM is detected on 3rd day, IgG on 7th day.

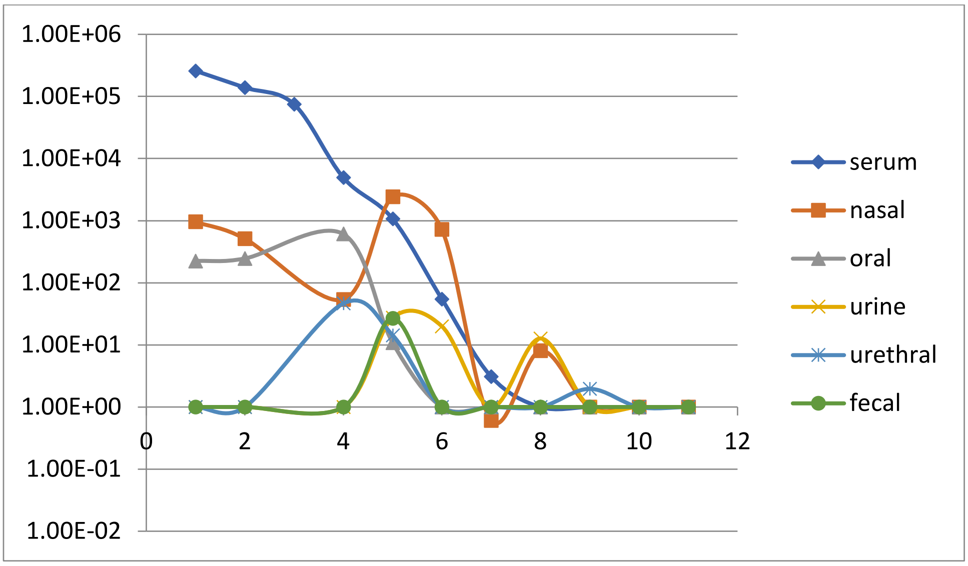

Figure S1: Y2 coded patient's viral load slope according to days

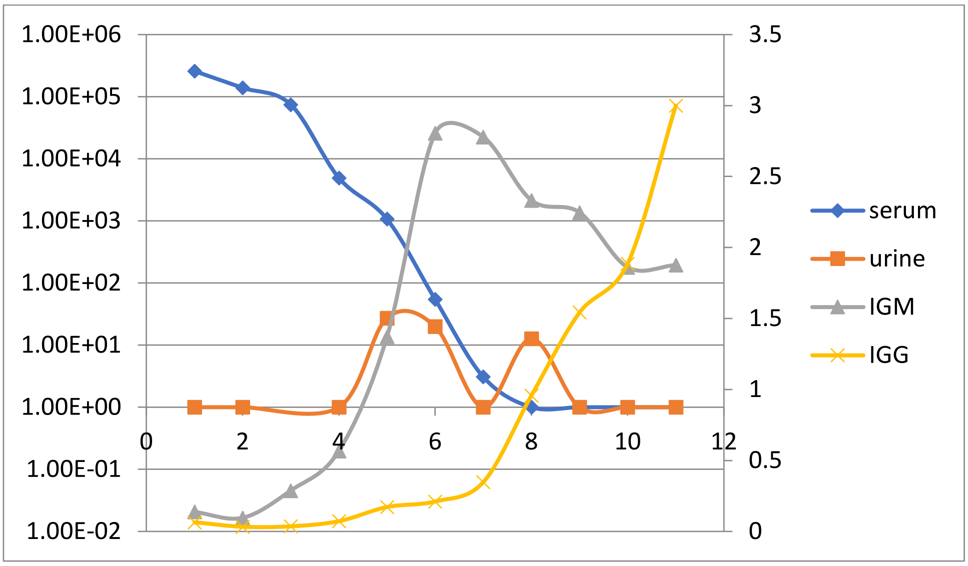

Figure S2: Y2 coded patient's antibody response and viral load slope according to days

	3	4	5	6	7	8	9	10	11	12	13
Serum*	1,59E+07	6,25E+05	3,19E+05	7,12E+03	4,07E+03	8,98E+03	2,00E+03	4,68E+03	2,22E+03	2,47E+05	1,46E+03
Nasal	4,50E+03	3,69E+01	2,03E+02	8,88E+02	Negative	Negative	Negative	Negative	N/A	Negative	Negative
Oral	1,43E+03	8,88E+00	4,03E+01	1,73E+02	Negative	Negative	Negative	Negative	Negative	Negative	Negative
Urine	4,75E+01	3,63E+00	8,90E+00	9,26E+01	5,38E+02	1,55E+03	1,49E+03	4,40E+03	3,92E+03	5,62E+02	Negative
Vaginal	2,01E+01	7,20E+01	2,86E+01	2,79E+02	8,58E+02	Negative	Negative	Negative	Negative	Negative	Negative
Fecal	Negative	Negative	Negative	2,38E+03	4,54E+01	Negative	Negative	Negative	N/A	Negative	Negative
IGM	0,018	0,042	0,158	1,549	1,961	3	3	3	2,412	0,728	1,482
IGG	0,073	0,146	0,153	0,191	0,222	0,223	0,286	0,301	0,443	0,478	0,589

Table-S3: Y3 coded patient (viral loads in copies/mL and OD values on monitored days) (N/A: not sent to the laboratory) *On the 12th day Anti-CCHFV IgM decreases and serum viral load increases. While serum viral load decreases, viral load of urine increases on the 4th to 11th day.

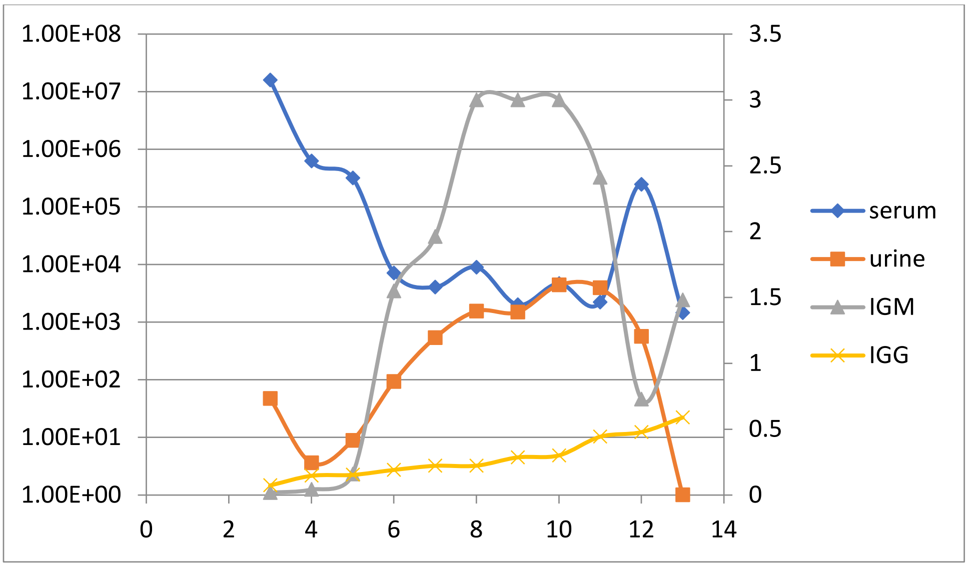

Figure S3: Y3 coded patient's antibody response and viral load slope according to days

	5	6	7
Serum	1,93E+03	1,69E+03	5,12E+03
Nasal	8,78E+02	Negative	3,18E+03
Oral	3,11E+03	5,40E+02	5,38E+03
Urine	1,85E+03	1,45E+03	7,64E+03
Vaginal	3,84E+03	1,43E+02	5,20E+03
Fecal*	Negative	Negative	2,06E+03
IGM	0,817	1,019	1,918
IGG	0,097	0,059	0,1

Table-S4: Y4 coded patient (viral loads in copies/mL and OD values on monitored days) *Fecal swab is positive on the 7th day, negative on the 5th and 6th day.

	4	5	6	7	8	9	10
Serum*	5,66E+04	1,57E+05	1,70E+05	3,47E+05	8,06E+05	3,18E+03	1,92E+03
Nasal	1,13E+03	Negative	3,70E+03	Negative	Negative	Negative	N/A
Oral	1,71E+03	5,89E+04	6,47E+04	1,00E+00	8,68E+03	Negative	N/A
Urine	2,82E+01	Negative	Negative	Negative	8,07E+02	2,31E+03	N/A
Urethral	6,85E+03	Negative	6,63E+03	3,31E+03	5,96E+03	4,76E+03	N/A
Fecal	Negative	3,90E+04	6,48E+03	Negative	Negative	Negative	N/A
IGM	0,081	0,316	0,298	0,92	1,602	1,636	1,438
IGG	0,031	0,032	0,078	0,122	0,182	0,211	0,423

Table-S5: Y5 coded patient (viral loads in copies/mL and OD values on monitored days) *While serum viral load decreases, viral load of urine increases on the 8th and 9th day.

	5	6	7	8	9	10	11	12
Serum	N/A	Negative	Negative	Negative	Negative	Negative	N/A	N/A
Nasal	Negative	Negative	Negative	Negative	Negative	Negative	N/A	N/A
Oral	Negative	Negative	Negative	Negative	Negative	Negative	N/A	N/A
Urine	N/A	Negative	Negative	Negative	Negative	Negative	N/A	N/A
Urethral	N/A	N/A	Negative	Negative	Negative	Negative	N/A	N/A
Fecal	Negative	Negative	Negative	Negative	Negative	Negative	N/A	N/A
IGM	1,218	1,632	1,743	1,733	2,151	1,876	1,922	1,179
IGG	0,072	0,057	0,063	0,068	0,168	0,164	0,199	0,06

Table-S6: Y6 coded patient who was started Ribavirin on the 1st day (viral loads in copies/mL and OD values on monitored days)

	6	7	8	9
Serum	1,57E+04	1,51E+01	1,38E+01	3,91E+00
Nasal	4,65E+03	1,45E+02	6,77E+01	Negative
Oral	2,43E+02	1,24E+02	Negative	Negative
Urine*	1,74E+01	Negative	1,01E+01	1,13E+01
Urethral	1,14E+02	9,39E+01	7,52E+02	3,28E+02
Fecal	1,21E+02	3,99E+01	3,60E+01	9,74E+00
IGM	1,624	2,297	2,013	1,828
IGG	0,09	0,222	0,526	1,616

Table-S7: N1 coded patient who was started Ribavirin on the 6th day (viral loads in copies/mL and OD values on monitored days) *While serum viral load decreases, viral load of urine stays at the same log value.

	6	7	8	9
Serum	2,23E+02	4,84E+00	6,37E+00	1,74E+00
Nasal	4,87E+00	6,10E+01	4,65E+00	1,19E+03
Oral	Negative	2,29E+00	Negative	Negative
Urine	1,39E+01	Negative	Negative	Negative
Vaginal	7,35E+01	Negative	Negative	Negative
Fecal	Negative	Negative	Negative	Negative
IGM	0,896	1,301	1,117	1,174
IGG	0,052	0,069	0,078	0,12

Table-S8: N2 coded patient who was started Ribavirin on the 6th day (viral loads in copies/mL and OD values on monitored days)

	3	4	5	6	7	8	9
Serum	Negative	Negative	Negative	Negative	Negative	Negative	Negative
Nasal	Negative	Negative	Negative	Negative	Negative	Negative	Negative
Oral	Negative	Negative	Negative	Negative	Negative	Negative	Negative
Urine	Negative	Negative	Negative	Negative	Negative	Negative	Negative
Fecal	Negative	Negative	Negative	Negative	Negative	Negative	Negative
IGM	0,026	0,067	0,391	1,099	1,654	1,846	N/A
IGG	0,047	0,05	0,051	0,066	0,083	0,112	N/A

Table-S9: C1 coded patient who was started Ribavirin on the 1st day (viral loads in copies/mL and OD values on monitored days)

	3	4	5	6	7	8	9
Serum	1,83E+02	4,91E+02	9,54E+03	Negative	Negative	N/A	Negative
Nasal	2,44E+01	1,00E+00	Negative	N/A	N/A	Negative	Negative
Oral	6,51E+01	Negative	Negative	Negative	Negative	Negative	Negative
Urine	Negative	Negative	Negative	N/A	Negative	Negative	Negative
Fecal	Negative	Negative	Negative	N/A	Negative	Negative	Negative
IGM	0,646	0,831	0,895	0,793	0,674	0,31	0,774
IGG	0,101	0,282	0,758	1,882	2,381	2,153	3

Table-S10: C2 coded patient who was started Ribavirin on the 1st day (viral loads in copies/mL and OD values on monitored days)

	4	5	6	7	8	9
Serum	Negative	Negative	Negative	Negative	Negative	Negative
Nasal	Negative	Negative	N/A	Negative	Negative	Negative
Oral	Negative	Negative	N/A	Negative	Negative	Negative
Urine	Negative	Negative	N/A	Negative	Negative	Negative
Fecal	1,72E+02	Negative	N/A	Negative	Negative	Negative
IGM	3	3	2,492	2,926	2,561	2,82
IGG	0,256	0,592	0,73	1,058	1,087	1,316

Table-S11: C3 coded patient who was started Ribavirin on the 1st day (viral loads in copies/mL and OD values on monitored days)

	5	6	7	8	9
Serum	6,51E+03	5,06E+03	2,12E+02	Negative	Negative
Nasal	Negative	Negative	Negative	Negative	Negative
Oral	8,03E+02	Negative	Negative	Negative	Negative
Urine	Negative	Negative	Negative	Negative	Negative
Urethral	Negative	N/A	Negative	Negative	Negative
Fecal	Negative	Negative	Negative	Negative	Negative
IGM	N/A	0,226	1,119	2,14	2,912
IGG	N/A	0,037	0,09	0,068	0,045

Table-S12: H1 coded patient who was started Ribavirin on the 4th day (viral loads in copies/mL and OD values on monitored days)

	7	8	9	10	11	12	13	14
Serum	4,59E+06	5,15E+05	1,58E+05	3,33E+03	2,35E+03	N/A	N/A	1,45E+03
Nasal	Negative	Negative	2,39E+03	3,02E+03	Negative	N/A	N/A	Negative
Oral	Negative	3,16E+04	7,96E+04	Negative	Negative	N/A	N/A	Negative
Urine	9,28E+01	2,41E+02	2,22E+02	1,30E+03	N/A	2,41E+03	N/A	8,76E+03
Vaginal	Negative	Negative	1,63E+03	4,35E+03	4,23E+03	N/A	N/A	Negative
Fecal	Negative	1,15E+04	1,37E+03	Negative	9,25E+02	N/A	N/A	Negative
IGM	0,392	1,053	2,025	2,435	N/A	2,292	N/A	1,595
IGG	0,025	0,04	0,047	0,102	N/A	0,093	N/A	0,355

Table-S13: H2 coded patient who was started Ribavirin on the 4th day (viral loads in copies/mL and OD values on monitored days) *Anti-CCHFV IgM increases and serum viral load decreases. While serum viral load decreases, viral load of urine increases on the 7th to 14th day.

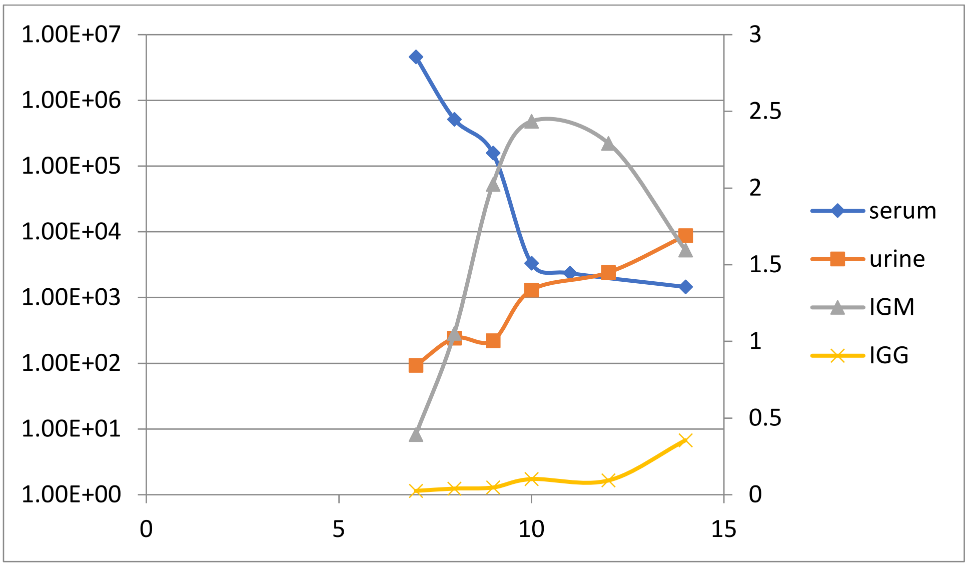

Figure S4: H2 coded patient's antibody response and viral load slope according to days

	3	4	5	6	7	8	9	10
Serum	3,43E+03	9,89E+02	Negative	Negative	N/A	N/A	N/A	Negative
Nasal	Negative	1,45E+02	Negative	Negative	N/A	N/A	N/A	Negative
Oral	2,02E+04	1,05E+03	Negative	Negative	N/A	N/A	N/A	Negative
Urine	Negative	Negative	Negative	Negative	N/A	N/A	N/A	Negative
Urethral	N/A	Negative	Negative	Negative	N/A	N/A	N/A	Negative
Fecal	Negative	Negative	Negative	Negative	N/A	N/A	N/A	Negative
IGM	1,728	1,323	1,834	1,716	N/A	N/A	N/A	1,034
IGG	0,093	0,156	0,216	0,616	N/A	N/A	N/A	1,254

Table-S14: H3 coded patient who was started Ribavirin on the 2nd day (viral loads in copies/mL and OD values on monitored days)

	10	11	12	13	14	15	16	17	18	19
Serum	3,53E+06	3,71E+05	1,09E+04	1,64E+03	1,20E+04	N/A	Negative	8,99E+03	Negative	Negative
Nasal	Negative	Negative	Negative	2,22E+03	2,49E+02	1,00E+00	4,81E+01	1,65E+02	Negative	Negative
Oral	7,20E+03	5,43E+04	6,65E+03	1,39E+03	1,96E+02	Negative	Negative	Negative	Negative	Negative
Sweat	Negative	Negative	Negative	Negative	Negative	7,97E+01	Negative	Negative	Negative	5,18E+01
Urine	1,49E+02	5,72E+01	Negative	Negative	Negative	Negative	Negative	Negative	Negative	Negative
Urethral	N/A	Negative	Negative	Negative	Negative	Negative	Negative	Negative	Negative	Negative
Fecal	4,71E+05	4,35E+04	1,17E+04	1,85E+03	4,32E+03	1,01E+03	Negative	1,02E+02	6,72E+01	Negative
IGM	1,075	1,521	3	3	3	3	3	3	3	N/A
IGG	0,024	0,03	0,102	0,851	3	3	3	3	3	N/A

Table-S15: H4 coded patient who was started Ribavirin on the 10th day (viral loads in copies/mL and OD values on monitored days)

	5	6	7	8	9	10	11	12	13
Serum	5,94E+05	2,33E+05	4,63E+04	2,97E+04	1,04E+04	3,33E+02	1,94E+03	5,83E+02	Negative
Nasal	Negative	7,57E+02	2,27E+04	4,02E+03	Negative	2,25E+02	8,36E+01	Negative	Negative
Oral	9,63E+04	5,19E+04	2,17E+03	Negative	Negative	Negative	Negative	Negative	Negative
Urine	3,05E+03	2,71E+03	9,48E+02	Negative	1,41E+03	Negative	1,45E+03	1,77E+03	1,04E+03
Vaginal	6,55E+02	5,60E+04	4,20E+04	1,29E+04	5,26E+04	3,60E+02	9,22E+02	1,29E+02	Negative
Fecal	3,02E+03	1,37E+03	4,82E+02	8,19E+02	4,34E+02	2,57E+02	2,10E+02	Negative	Negative
IGM	0,351	0,608	2,277	3	3	3	3	3	3
IGG	0,026	0,023	0,082	0,107	0,113	0,169	0,214	0,274	0,324

Table-S16: H5 coded patient who was started Ribavirin on the 5th day had reversible renal failure, vaginal bleeding but not hematuria nor gastrointestinal bleeding (viral loads in copies/mL and OD values on monitored days) *While serum viral load decreases, viral load of urine is on the same log, on the 11th to 13th day.

	9	10	11	12	13	14	15	16	17
Serum	2,25E+02	Negative	Negative	Negative	Negative	Negative	Negative	Negative	Negative
Nasal	4,78E+01	Negative	Negative	Negative	Negative	Negative	Negative	Negative	Negative
Oral	1,96E+02	Negative	Negative	Negative	Negative	Negative	Negative	Negative	Negative
Urine	2,73E+04	7,19E+03	6,01E+04	2,10E+03	1,27E+04	2,17E+03	Negative	3,58E+03	Negative
Urethral	Negative	8,06E+02	Negative	1/4,90E+03	Negative	Negative	Negative	Negative	Negative
Fecal	Negative	Negative	Negative	Negative	Negative	Negative	N/A	Negative	Negative
IGM	2,128	1,726	1,404	1,305	1,386	0,967	0,855	0,791	0,552
IGG	1,345	2,071	1,906	1,821	1,437	1,814	2,054	2,39	3

Table-S17: H6 coded patient (viral loads in copies/mL and OD values on monitored days) *While serum CCHFV-RNA becomes negative, viral load of urine is on the same log, on the 11th to 14th day and positive on the 16th day.

	5	6	7	8	9	10	11	12	13
Serum	1,17E+05	2,02E+04	6,00E+03	5,46E+03	8,53E+02	Negative	Negative	Negative	Negative
Nasal	Negative	7,08E+02	2,41E+02	3,69E+02	2,26E+02	3,47E+02	5,88E+01	2,02E+02	Negative
Oral	1,97E+02	4,53E+03	3,88E+02	Negative	Negative	Negative	Negative	Negative	Negative
Urine	4,74E+04	1,45E+03	8,16E+04	8,76E+04	3,89E+03	2,76E+04	6,77E+03	1,67E+04	5,88E+04
Urethral	Negative	5,81E+02	Negative	N/A	Negative	Negative	Negative	4,54E+02	Negative
Fecal	1,32E+03	4,79E+04	1,21E+04	N/A	5,87E+03	Negative	Negative	Negative	Negative
IGM	1,591	2,27	2,335	2,325	2,151	1,746	1,724	1,695	1,645
IGG	0,02	0,051	0,096	0,186	0,396	0,405	0,461	0,72	1,252

Table-S18: H7 coded patient had reversible renal failure (viral loads in copies/mL and OD values on monitored days) *While serum CCHFV-RNA decreases and becomes negative on the 10th day, viral load of urine is on the same log ranges, on the 10th to 13rd day.

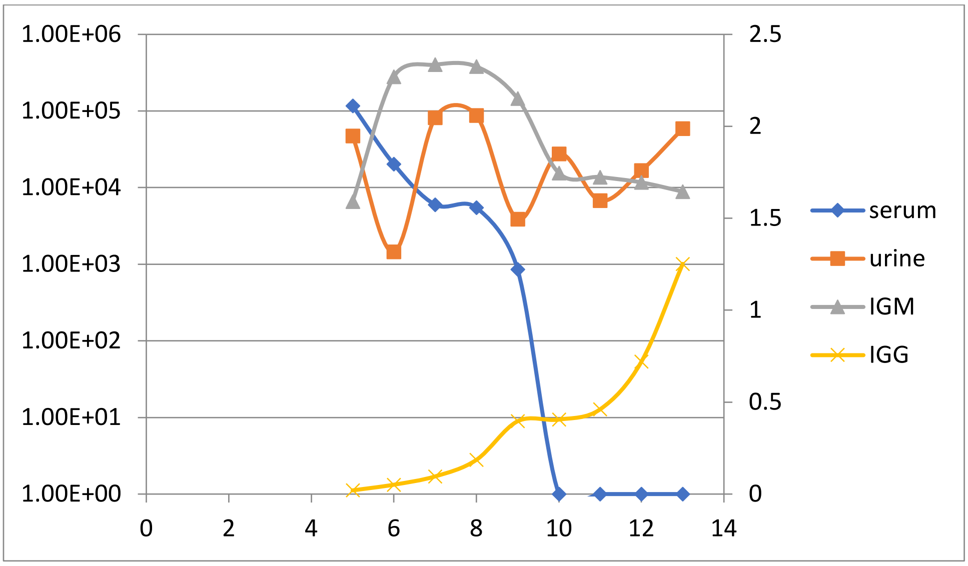

Figure S5: H7 coded patient's antibody response and viral load slope according to days
